# Itraconazole Niosomes Drug Delivery System and Its Antimycotic Activity against *Candida albicans*

**DOI:** 10.5402/2012/653465

**Published:** 2012-12-13

**Authors:** Vijay D. Wagh, Onkar J. Deshmukh

**Affiliations:** ^1^Department of Pharmaceutics, R. C. Patel Institute of Pharmaceutical Education and Research, Maharashtra, Shirpur 425405, India; ^2^Formulation and Development Department, Shreya Life Sciences Pvt. Ltd., MIDC, Waluj, Maharashtra, Aurangabad 431001, India

## Abstract

Niosomes have potential applications in topical drug delivery system. The objective of the study was to formulate and evaluate the niosome of Itraconazole. Surfactant : cholesterol ratio and quantity of ethanol used were studied by applying factorial design. Formulated niosomes were evaluated for vesicle size, entrapment efficiency, drug release, skin permeation, and antimycotic activity. Vesicle size, entrapment efficiency, and drug release were markedly dependent on surfactant : cholesterol ratio and quantity of ethanol used. Permeation of the drug through the skin was affected by cholesterol content in formulation. Itraconazole niosome were having larger zone of inhibition than marketed formulation when activity was checked against *C. albicans*. Niosomes may be a promising carrier for topical delivery of Itraconazole especially due to their simple production.

## 1. Introduction

Most antifungal drug substances are lipophilic compounds, which are practically insoluble in water [1]. For skin care and the topical treatment of dermatological disease, a wide choice of vehicles ranging from solids to semisolids and liquid preparations is available to clinicians and patients [2]. Topical application of antimicrobial agents is a useful tool for the therapy of skin and soft-tissue infections [2]. A number of strategies to deliver antifungal using nanocarriers are developed to facilitate drug targeting infected cells. Nanosized carriers have been receiving special attention with the aim of minimizing the side effects and improving efficacy of drug therapy. Several nanosized delivery systems have already proved their effectiveness in antifungal therapy [3]. The total therapeutic effect of percutaneous preparations depends not only on the action of the drug itself, but also on other factors related to the structure of the vehicle. Taking into account the peculiarities of fungal parasites, the focus is placed particularly on lipid-based vehicles, and earlier studies have shown that results in improved antifungal activity [4]. Many techniques have been aimed to disrupt and weaken the highly organized intercellular lipids in an attempt to enhance drug transport across the intact skin; one of them is the vesicle formulation as skin delivery system [5–8].

Niosomes have been recognized as a good vehicles for the topical delivery of drugs [9]. They serve as “organic” solvent for the solubilization of poorly soluble drugs, for instances corticosteroids; as a result, higher local drug concentrations at the maximum thermodynamic activity can be applied. They may serve as a local depot for the sustained release of dermal active compounds including antibiotics, corticosteroids, or retinoic acid. By virtue of penetration of individual phospholipid molecules or nonionic ether surfactants into the lipid layers of the stratum corneum and epidermis, they may serve as penetration enhancer and facilitate dermal delivery leading to higher localized drug concentrations. They may serve as rate-limiting membrane barrier for the modulation of systemic absorption, that is, they may serve as controlled transdermal delivery systems. 

Mainly two types of vesicle skin interactions occurs during *in vitro* studies using human skin which may induce various effects on dermal or transdermal drug delivery [10–12]. First, the vesicles in contact with stratum corneum aggregate fuse and adhere to the cell surface. It is believed that this interaction leads to a high thermodynamic activity gradient of the drug at the vesicle stratum corneum interface, which is the driving force for penetration of the lipophilic drugs across the stratum corneum. Secondly, this type of interaction involves the ultrastructural changes of the intercellular lipid regions of the stratum corneum and its deeper layers at maximum depth of about 10 *μ*m as revealed by freeze fracture electron microscopy (FFEM) and Small Angle X-ray Scattering (SAXS).

Itraconazole is synthetic triazole and 1 : 1 : 1 : 1 racemic mixture of four diastereoisomers (two enantiomeric pairs), each possessing 3 chiral centres. The structural formula is closely related to the imidazole and ketoconazole. Itraconazole is a drug of choice for patients with indolent, nonmeningeal infections due to *B. dermatitidis, H. capsulatum, P. brasiliensis,* and *C. immitis.* Approximately half of the patients with distal subungual onychomycosis respond well to Itraconazole. Itraconazole is often the best choice for the treatment of pseudallescheriasis, an infection not responding to the amphotericin B therapy, as well as cutaneous or extracutaneous sporotrichosis, tinea corporis, and extensive tinea versicolor. Itraconazole is used in the treatment of toenail onychomycosis with terbinafine as one week per month for three months. Itraconazole has low solubility and low permeation. By incorporation of Itraconazole in small niosomes, the drug can be targeted directly to the site of action, thus enhancing its therapeutic efficacy.

## 2. Materials and Methods

### 2.1. Materials

Itraconazole was received as a gift sample from USV Ltd., Mumbai, India. Span 60 was purchased from Loba Chemie Private Ltd. Cholesterol AR (Mol. Wt.—386.6 D) was purchased from Qualigens fine chemicals Ltd., India. Ethanol Propanol were obtained from Research Lab Chem, Mumbai. Other chemicals were pharmacopeial grade or A.R. grade. Fresh distilled water was used for all the formulation.

### 2.2. Formulation of Niosomes

Niosomes were prepared by hydration of proniosome [13]. Precisely, surfactants : cholesterol (total surfactant 500 mg) and drug (50 mg) were taken in a clean and dry, wide mouth small glass vial. The surfactants : cholesterol ratio and quantity of ethanol used in formulation were as per the factorial design. Compositions of 9 batches of niosomes are given in Table 1. After mixing all the ingredients, the open end of the glass tube was covered with a lid to prevent loss of solvent from it and warmed on a water bath at 60–70°C for about 5 min, until the surfactants were dissolved completely. The aqueous phase (0.1% glycerol solution) was added and warmed on a water bath till clear solution is formed on cooling converts into niosomal formulation. The dispersion obtained was preserved in same glass tube in dark for characterization.

### 2.3. Size, Shape, and Morphological Characterization [14]

Vesicular structure of surfactant-based vesicles can be visualized by microscope. Size of the niosomes prepared from proniosomes can be analyzed by 3 different method, namely, agitations (shaking) without agitation and with sonication. Optical microscope with digital camera (catcham) was used to study size, shape, and morphology of niosomes.

### 2.4. Entrapment Efficiency [14]

 The Analysis of entrapment efficiency can be done by centrifugation method. The niosome entrapped drug was separated from the free drug by the centrifugation method. The prepared niosomal dispersion was subjected for centrifugation at high 7000 rpm for 30 min. Clear supernatant liquid was separated from niosomes. Niosomes were disrupted and analyzed by using spectrophotometer to calculate the amount of entrapped drug:

(1) $$\begin{array}{c} \text{Percent}\;\text{entrapped}=\left[\frac{\text{Entrapped}\;\text{drug(mg)}}{\text{Total drug}\;\text{added(mg)}}\right]\times 100. \end{array}$$

### 2.5. *In Vitro* Drug Release

Drug release from niosome was tested with modified Keshary-Chien diffusion cell (receptor compartment capacity 20 mL), using cellophane membrane with permeation area of 2.54 cm^2^. 20 mL of release medium (15 mL 7.4 phosphate buffer and 5 mL methanol) was added to the acceptor chamber. The prepared niosomal dispersion was subjected for centrifugation at high 7000 rpm for 30 min to separate the unentrapped drug. Niosomes encapsulating drug equivalent to 50 mg was placed in donor compartment. The entire assembly was kept on a magnetic stirrer and was maintained at 37°C ± 1°C. At predetermined time points, 1 mL samples were withdrawn from the acceptor compartment, replacing the sampled volume with release medium after each sampling for a period of 6 hrs. The samples were suitably diluted with solvent (chloroform 1 : 4 v/v and filled up with methanol 3 : 4 v/v) and measured spectrophotometrically at 260 nm.

### 2.6. *In Vitro* Skin Permeation Study [9]

The Wistar rat was given free access to food and water 24 hr. The hair was removed by hair clipper. The rat was killed by respiratory paralysis. The abdominal skin was carefully excise and fatty layer, debris, was carefully removed. The skin was washed with saline solution and used within 24 hr. The skin was mounted on Modified Keshary-Chien diffusion cell with permeation area of 2.54 cm^2^ (receptor compartment capacity 20 mL) with stratum corneum facing donor compartment. 20 mL of release medium (15 mL 7.4 phosphate buffer and 5 mL methanol) was added to the acceptor chamber. The prepared niosomal dispersion was subjected for centrifugation at high 7000 rpm for 30 min to separate the unentrapped drug. Niosomes encapsulating drug equivalent to 50 mg was placed in donor compartment. Niosomes encapsulating drug equivalent to 50 mg was placed in donor compartment. The entire assembly was kept on a magnetic stirrer and was maintained at 37°C ± 1°C. At predetermined time points, 1 mL samples were withdrawn from the acceptor compartment, replacing the sampled volume with release medium after each sampling for a period of 8 hrs. The samples were suitably diluted with solvent (chloroform 1 : 4 v/v and filled up with methanol 3 : 4 v/v) and measured spectrophotometrically at 260 nm.

### 2.7. *In Vitro* Antimycotic Study [15]

Agar-cup diffusion method was adopted. These tests were carried out using cultures of *Candida albicans *(AJ 005123) (0.1%), in Sabouraud dextrose agar. Active culture of *Candida albicans *strain was inoculated in sterile 0.85% NaCl tube in a ratio of 1 : 9. Further dilution of the culture where prepared in a sterile 0.85% NaCl to get 10^6^ CFU/mL. Seeding of culture performed by swabbing method in which sterile swab was dipped into the culture suspension and excess fluid removed by pressing gently against the wall of test tube. Swab was placed on the edge of the agar plate and move across to the other sides, this was separated to obtain an even spread in sterile conditions. Using borer, wells were made in the seeded agar plates of 6 mm diameter and to it ES60A to ES60I encapsulating 20 mg of itraconazole and 200 mg of marketed formulation (1%) were added. Plates were kept in freezer for diffusion for 10–15 min. And then placed in incubator for incubation period of 24–48 hr at 37°C. Results were seen as diameter of zone of inhibition and compared with that of empty niosomal formulation and marketed formulation (Itral).

## 3. Results and Discussions

The method of preparation of proniosomes is based on the simple idea that the mixture of surfactant:alcohol:aqueous phase can be used to form the concentrated proniosomal gel, which can be converted to a stable niosomal dispersion by dilution with excess aqueous phase. This technique involves the principle of coacervation-phase separation [14].

### 3.1. Vesicle Characteristics

Optical microscope with digital camera (catcham) was used to study size, shape, and morphology of niosomes. Vesicles were found to be spherical, discrete, and multilammelar. Results are represented in Table 2. Size of vesicles was found to be in the order of hydration: without agitation > with agitation > with ultrasonication. Batch ESC60C (35.88 ± 1.96 *μ*m) has shown the largest size vesicles were formed after hydration of proniosomes without agitation. Whereas, ES60 G (16.67 ± 1.76 *μ*m) with shaking and ES60 G (12.46 ± 0.78 *μ*m) after sonication (Ultrasonic cleaner, Trans-o-sonic, Lab Hosp, Mumbai) has shown the smaller vesicles. This might be due to energy applied during agitation, and ultrasonication results in their breakage into smaller vesicles; ultrasonication energy being highest results in small unilamellar vesicles [14]. These results states that increase in cholesterol content increases hydrophobicity. Increased hydrophobicity decreases surface energy which subsequently reduces vesicle size [16–20]. The polydispersity index was always found to be very low, showing that formulated niosome are uniform in size [14].

### 3.2. Entrapment Efficiency (%)

Table 3 represents entrapment efficiency % of various formulation batches. With increasing cholesterol, the bilayer hydrophobicity and stability increased [21, 22] and permeability decreased [23] which lead to efficiently trapping the hydrophobic drug into bilayers as vesicles formed. Entrapment efficiency was decreased with increase in cholesterol ratio above a certain limit may be due to the fact that increasing cholesterol beyond a certain concentration can disrupt the regular linear structure of vesicular membranes [24]. Entrapment efficiency has an impact on solubility of drug in the alcohol. Entrapment efficiency increased with the increase in alcohol quantity.

### 3.3. *In Vitro *Drug Release

**Figure 1:**
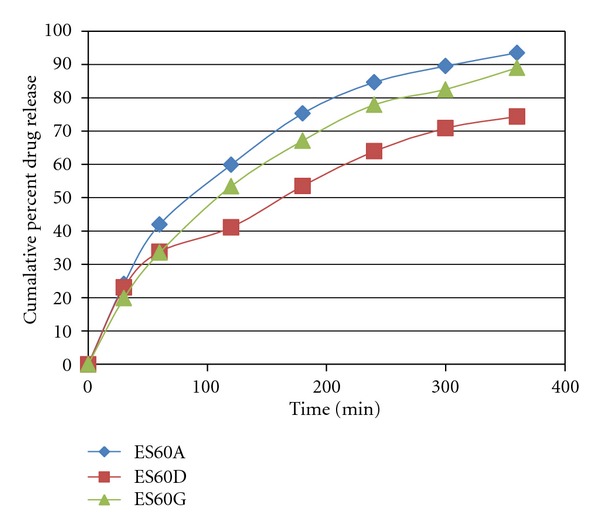
*In vitro* drug release of Itraconazole-loaded niosomes formulation of ES60A, ES60D, and ES60G.

**Figure 2:**
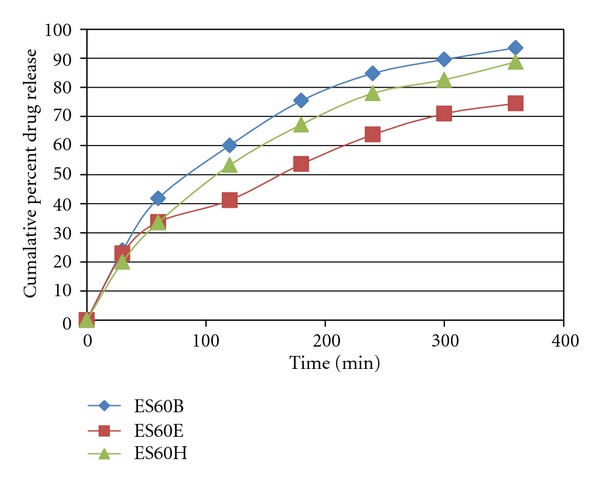
*In vitro* drug release of Itraconazole-loaded niosomes formulation of ES60B, ES60E, and ES60H.

**Figure 3:**
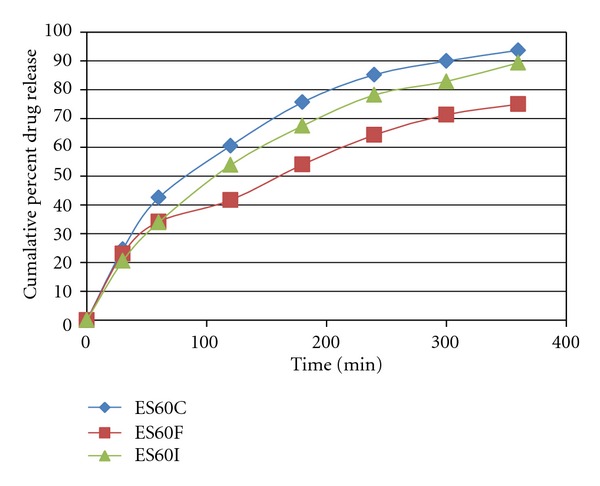
*In vitro* drug release of Itraconazole-loaded niosomes formulation of ES60C, ES60F, and ES60I.

*In vitro* Drug release was represented by Figures 1, 2, and 3. The rate of release was in the order ES60D < ES60E < ES60F < ES60H < ES60G < ES60I < ES60A < ES60B < ES60C. Increasing the cholesterol content resulted in more intact lipid bilayers as a barrier for drug release and decreased its leakage by improving the fluidity of the bilayer membrane and reducing its permeability, which led to lower drug elution from the vesicles. When the cholesterol content increased above a certain limit, the release rate increased due to the fact that increasing cholesterol beyond a certain concentration can disrupt the regular linear structure of vesicular membranes [24, 25].

### 3.4. Multiple Regression Analysis of Factorial Design

**Figure 4:**
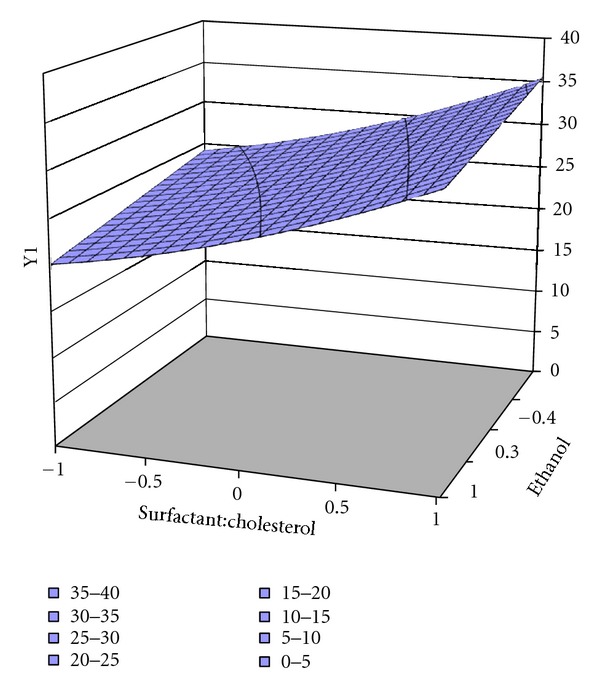
Surface response plot for response Y1 of ES60, vesicle size.

**Figure 5:**
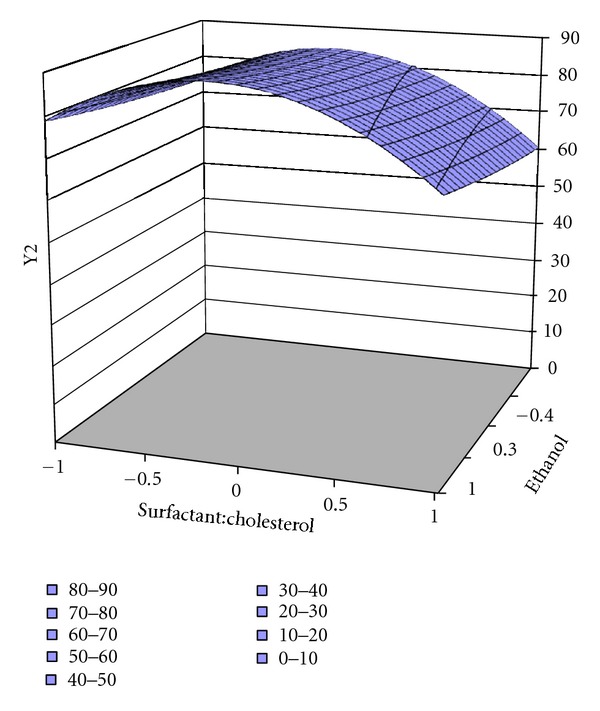
Surface response plot for response Y2 of ES60, entrapment efficiency %.

**Figure 6:**
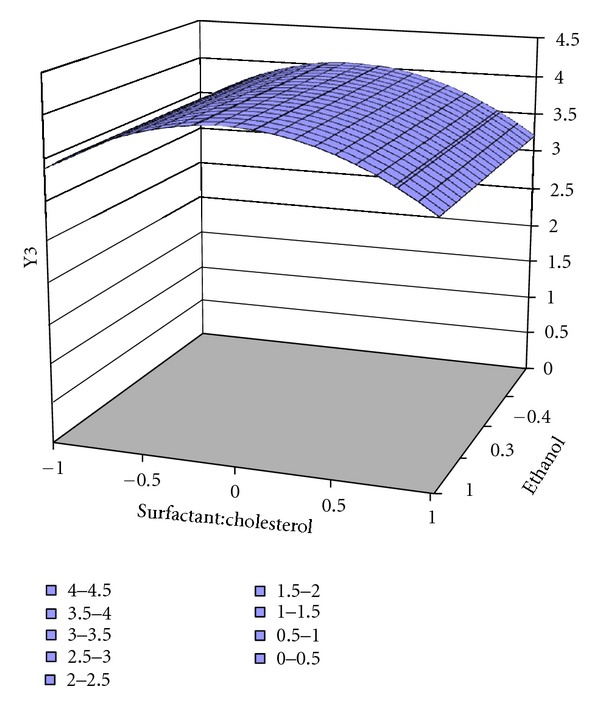
Surface response plot for response Y3 of ES60, drug release.

The responses of factorial design Y1 (vesicle size) and Y2 (encapsulation efficiency %) were subjected to multiple regression analysis by PCP DISSO software. Surface response plots (as shown in Figures 4, 5, and 6) and coefficient values indicate both independent variables X1 (surfactant : cholesterol) and X2 (quantity of alcohol used) affect responses (Tables 4 and 5).

### 3.5. Skin Permeation Studies

**Figure 7:**
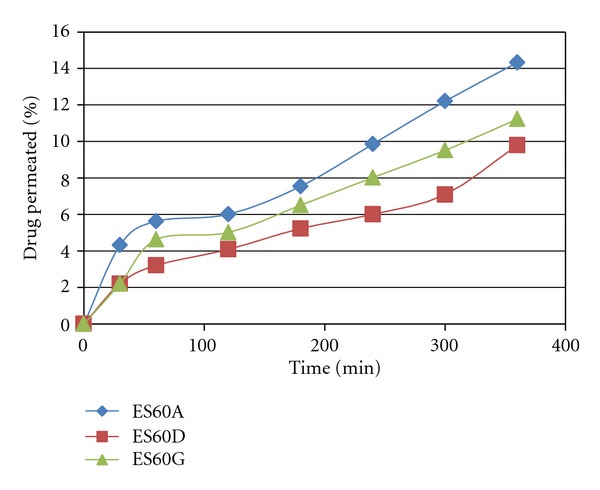
Percent of drug permeated from ES60A, ES60D, and ES60G.

Figure 7 shows the percent of drug permeated after 6 hr through skin form; it was in the order ES60A > ES60G > ES60D. Increasing the cholesterol content resulted in decreased permeation of Itraconazole. When the cholesterol content increased above certain limit, permeation is increased due to the fact that increasing cholesterol beyond a certain concentration can disrupt the regular linear structure of vesicular membranes [25–27]. Amount of alcohol used in formulation has no significant effect on permeation.

### 3.6. Antimycotic Study of Formulated Niosomes

**Figure 8:**
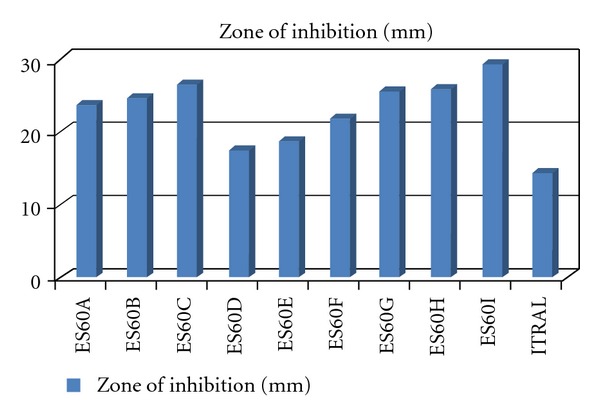
Zone of inhibition (mm) of ES60A-ES60I, and Marketed formulation (Itral).

Figure 8 represents zone of inhibition of formulated niosome batches and marketed formulation (Itral). Zone of inhibition of niosome formulatio showed that they are more (90 × effective than marketed formulation (Itral)). The empty niosomal formulation was not shown in the zone of inhibition and antimycotic action.

## 4. Conclusion

Formulated niosomes were evaluated foe vesicle size, entrapment efficiency, drug release, skin permeation, and antimycotic activity. Vesicle size, entrapment efficiency, and drug release were dependent on surfactant : cholesterol ration and quantity of ethanol used. Formulated niosomes were having a good skin permeation and were more effective in antimycotic activity when compared against marketed formulation. Niosome may be a promising carrier for topical delivery of Itraconazole.

## Figures and Tables

**Table 1:** Composition of niosome formulation.

Batch code	Surfactant : cholesterol molar ratio	Ethanol (mL)	Drug (mg)
ES60A	7 : 3	1.5	50
ES60B	7 : 3	1	50
ES60C	7 : 3	0.5	50
ES60D	6 : 4	1.5	50
ES60E	6 : 4	1	50
ES60F	6 : 4	0.5	50
ES60G	5 : 5	1.5	50
ES60H	5 : 5	1	50
ES60I	5 : 5	0.5	50

**Table 2:** Vesicle size of niosomes.

Batch code	Size of niosome in *μ*m					
	Without shaking VS ± SD*	PI*	With shaking VS ± SD	PI	After sonication VS ± SD	PI
ES60A	31.76 ± 1.41	0.04	28.27 ± 1.52	0.05	22.29 ± 1.96	0.08
ES60B	33.19 ± 1.42	0.04	30.17 ± 1.98	0.06	23.41 ± 1.52	0.06
ES60C	35.88 ± 1.96	0.05	31.39 ± 0.96	0.03	25.82 ± 2.22	0.08
ES60D	24.10 ± 1.49	0.06	21.30 ± 1.06	0.05	16.02 ± 1.35	0.08
ES60E	26.38 ± 1.52	0.05	23.84 ± 1.79	0.07	17.06 ± 1.16	0.06
ES60F	29.00 ± 1.22	0.04	25.55 ± 1.16	0.04	21.20 ± 1.17	0.05
ES60G	20.13 ± 2.33	0.11	16.67 ± 1.76	0.10	12.46 ± 0.78	0.06
ES60H	22.31 ± 2.30	0.10	19.96 ± 2.19	0.11	13.15 ± 0.89	0.06
ES60I	23.90 ± 1.54	0.06	20.65 ± 1.78	0.08	14.92 ± 1.42	0.09

*Mean of three separate observations ± standard deviation.

SD: standard deviation.

VS: mean vesicle size in *μ*m.

PI: polydispersity index obtained as PI = (SD/VS).

**Table 3:** Entrapment efficiency of Itraconazole-loaded noisome.

Sr. no.	Batch code	Entrapment efficiency (%)
1	E60A	68.77 ± 2.28
2	E60B	64.61 ± 1.61
3	E60C	60.79 ± 1.19
4	E60D	89.67 ± 1.85
5	E60E	85.28 ± 1.29
6	E60F	82.81 ± 0.56
7	E60G	78.16 ± 1.35
8	E60H	75.30 ± 1.21
9	E60I	73.06 ± 2.07

**Table 4:** Responses subjected to multiple regression analysis.

Response	Factorial batches								
	ES60A	ES60B	ES60C	ES60D	ES60E	ES60F	ES60G	ES60H	ES60I
Vesicle size (*μ*m) (Y1)	31.76	33.19	35.88	24.10	26.38	29.00	20.13	22.31	23.90
Entrapment efficiency (%) (Y2)	68.77	64.61	60.79	89.67	85.28	82.81	78.16	75.30	73.06
Time required for 50% drug release (hr) (Y3)	3.20	3.20	3.20	4.03	4.02	4.00	3.37	3.37	3.35

**Table 5:** Coefficient values obtained from multiple regression analysis.

Responses studied	Coefficients for ES60						
	bo	b1	b2	b11	b12	b21	*R* ^2^
Vesicle size (*μ*m) (Y1)	26.49	5.74	−2.13	1.36	—	—	0.9970
Entrapment efficiency (%) (Y2)	85.44	−5.50	3.32	0.69	−15.92	0.71	0.9997
Time required for 50% drug release (hr) (Y3)	4.01	−0.08	—	−0.73	—	—	0.999
